# MR elastography-derived right ventricular myocardial stiffness in dogs with congenital pulmonary valve stenosis: correlation with myocardial relaxation times and ECV

**DOI:** 10.1186/1532-429X-16-S1-P82

**Published:** 2014-01-16

**Authors:** Juliana Serafim da Silveira, Brian A Scansen, Peter A Wassenaar, Brian Raterman, Ning Jin, Richard D White, John D Bonagura, Arunark Kolipaka

**Affiliations:** 1 Department of Radiology, The Ohio State University College of Medicine, Columbus, Ohio, USA; 2 Department of Veterinary Clinical Sciences-College of Veterinary Medicine, The Ohio State University College of Medicine, Columbus, Ohio, USA; 3 Siemens Medical Solutions, Malvern, Pennsylvania, USA; 4 Department of Internal Medicine-Division of Cardiovascular Medicine, The Ohio State University College of Medicine, Columbus, Ohio, USA

## Background

Cardiac magnetic resonance elastography (CMRE) is a novel imaging technique to noninvasively quantify myocardial stiffness. Previous studies have demonstrated that excess interstitial fluid or fibrosis causes increased myocardial stiffness and also alter T1, T2 relaxation times, and myocardial extracellular volume fraction (ECV). Nonetheless, T1, T2 and ECV have not yet been correlated with either invasive or noninvasive measures of myocardial stiffness. The aim of our study was to demonstrate the feasibility of quantifying right ventricular free wall (RVFW) stiffness using CMRE and correlate it with intrinsic myocardial relaxation times and ECV in dogs with severe congenital pulmonary valve stenosis causing RV hypertrophy.

## Methods

In-vivo CMRE was performed on six dogs using a 1.5T scanner (Avanto, Siemens Healthcare). A basal RV short-axis slice was acquired using GRE-MRE, T1-Molli and T2 prepared B-SSFP sequences. Wave images were processed using MRE Lab (Mayo Clinic, Rochester, MN) to obtain end-systolic and end-diastolic stiffness maps. Regions of interest (ROIs) were drawn to determine RVFW effective stiffness, T1 and T2 values. Blood pool T1 values were obtained by placing ROIs in the center of the RV. Pre-contrast T1 values were corrected for varying heart rate. ECV was calculated using the formula: (ΔR1 myocardium/ΔR1 blood) × (1-hematocrit). Least squares linear regression was performed to determine the correlation between end-systolic and end-diastolic RVFW stiffness against ECV, T1 and T2 relaxation times.

## Results

**Figure 1:**
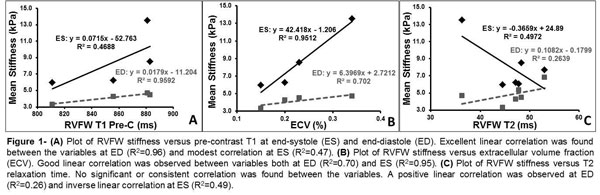
**(A) Polot of RVFW stiffness versus pre-contrast T1 at end-sysole (ES) and end-diastole (ED)**. Excellent linear correlation was found between the variables at ED (R^2 ^= 0.96) and modest correlation at ES (R^2 ^= 0.47). (B) Plot of RVFW stiffness versus extracellular volume fraction (ECV). Good linear correlation was observed between variables both at ED (R^2 ^= 0.70) and ES (R^2 ^= 0.95). (C) Plot of RVFW stiffness versus T2 relaxation time. No significant or consistent correlation was found between the variables. A positive linear correlation was observed at ED (R^2 ^= 0.26) and inverse linear correlation at ES (R^2 ^= 0.49).

RVFW myocardial effective stiffness in all animals was higher at end-systole (5.9 to 13.5 kPa) compared to end-diastole (3.3 to 6.8 kPa). RVFW native T1, T2 and ECV values ranged from 811 to 883 ms, 36 to 53 ms and 15% to 34%, respectively. Positive correlations were found between effective stiffness versus T1 and ECV (R2 values ranging from 0.48-0.95) in four dogs (Figures 1A, B). T2 relaxation times did not correlate with RVFW stiffness (Figure 1C).

## Conclusions

This study demonstrates the feasibility of measuring effective RVFW stiffness using CMRE in the setting of RV hypertrophy. The correlation between T1, ECV and RVFW stiffness, may potentially suggest interdependence between changes in extracellular matrix and the mechanical properties of the heart. The absence of correlation between effective stiffness and T2 can be attributed to an absence of acute myocardial injury with edema in this specific cohort of dogs presenting chronic pressure overload.

## Funding

Department of Radiology, The Ohio State University College of Medicine.

